# Nucleus Mechanosensing in Cardiomyocytes

**DOI:** 10.3390/ijms241713341

**Published:** 2023-08-28

**Authors:** Isabella Leite Coscarella, Maicon Landim-Vieira, Hosna Rastegarpouyani, Prescott Bryant Chase, Jerome Irianto, Jose Renato Pinto

**Affiliations:** 1 Department of Biomedical Sciences, Florida State University, Tallahassee, FL 32306, USA; 2 Department of Biological Science, Florida State University, Tallahassee, FL 32306, USA; 3 Institute for Molecular Biophysics, Florida State University, Tallahassee, FL 32306, USA

**Keywords:** cardiomyocytes, mechanotransduction, cardiomyopathy

## Abstract

Cardiac muscle contraction is distinct from the contraction of other muscle types. The heart continuously undergoes contraction–relaxation cycles throughout an animal’s lifespan. It must respond to constantly varying physical and energetic burdens over the short term on a beat-to-beat basis and relies on different mechanisms over the long term. Muscle contractility is based on actin and myosin interactions that are regulated by cytoplasmic calcium ions. Genetic variants of sarcomeric proteins can lead to the pathophysiological development of cardiac dysfunction. The sarcomere is physically connected to other cytoskeletal components. Actin filaments, microtubules and desmin proteins are responsible for these interactions. Therefore, mechanical as well as biochemical signals from sarcomeric contractions are transmitted to and sensed by other parts of the cardiomyocyte, particularly the nucleus which can respond to these stimuli. Proteins anchored to the nuclear envelope display a broad response which remodels the structure of the nucleus. In this review, we examine the central aspects of mechanotransduction in the cardiomyocyte where the transmission of mechanical signals to the nucleus can result in changes in gene expression and nucleus morphology. The correlation of nucleus sensing and dysfunction of sarcomeric proteins may assist the understanding of a wide range of functional responses in the progress of cardiomyopathic diseases.

## 1. Introduction

Mechanotransduction is an important biological mechanism that regulates cellular responses and morphological adaptations [1]. Living cells exposed to mechanical stimuli present such crucial responses to continuous environmental dynamics by adaptation of biological functions. Mechanically induced changes are involved in various cellular events such as migration, survival, transformation, and invasion [2]. The ability to respond to mechanical forces is associated with mechano-sensing proteins that are capable of detecting mechanical cues, and the transmission of biochemical signals via intracellular processes that travel through the cells [3]. While the recognition that cells can sense forces and respond to mechanical stimuli is not new to the scientific community [4], a growing number of studies have shown an association of these mechanical responses with processes in the cell nucleus [5].

The nucleus is a mechanoresponsive organelle, which reacts to forces transmitted through the cytoskeleton, and translates into specific cellular responses [6,7,8]. These cellular responses can dictate not only morphological changes, but also change the cellular behavior by altering transcriptional machinery and cell fate regulation [9,10]. The mechanotransduction of externally applied forces is crucial for many mammalian cells, such as endothelial cells under sheer stress in blood vessels [11], the maturation and remodeling of bones [12], and even protein synthesis regulation in muscle mass [13]. However, internally generated forces could also be important for sensing and responding to biochemical signaling. This is particularly true for muscle cells, such as the heart’s cardiomyocytes, that are specialized with highly organized structures—microscopic sarcomeres comprising arrays of thick and thin filaments—which generate pressure and shorten, enabling macroscopic pumping of the blood in the cardiovascular system. The contraction–relaxation cycles of cardiomyocytes continue incessantly throughout an individual’s life, responding to physical and energetic variations, and aging. Internal mechanical cues generated by the continuous sarcomere contraction–relaxation cycles can be associated with intracellular mechano-sensing and transduction in cardiomyocytes.

**Figure 1:**
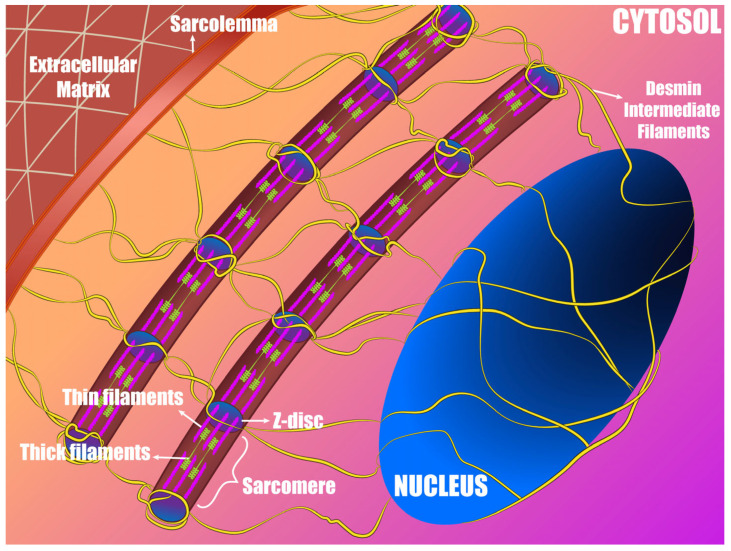
Schematic of intermediate filaments of desmin interconnected with sarcomeres and nucleus in a cardiomyocyte.

The ability to respond to and propagate signals received by the environment or intracellular forces are ensured by the cytoskeleton [14]. The cytoskeletal architecture is a network of filamentous proteins such as actin, microtubules, and intermediate filaments, which all connect to cytoplasmic proteins, membranes, and organelles, including the nucleus. The nucleus has a direct relation not only to the extracellular matrix (ECM), but also to the most important contractile mechanical unit of the muscle, the sarcomere [15,16]. Therefore, cardiomyocyte nuclei are not only capable of sensing mechanical stimuli from the environment, but also from the contractile sarcomeric apparatus (Figure 1).

This review focuses on the molecular alterations that can be found in cardiomyocyte nuclei during pathological processes such as cardiomyopathies. Genetic variants of sarcomeric proteins are primarily associated with the development of numerous myopathies, and over 1000 disease-related variants have been reported that are associated with cardiac contraction [17,18,19,20,21]. Although studies of pathological manifestations in cardiac muscle have advanced, and many phenotypic expressions have been elucidated and described as cardiomyopathies in recent years [22], not much is known about nuclear remodeling and mechanosensing in cardiomyopathies. Altered mechanical stimuli due to mutated sarcomeric proteins could trigger a wide range of functional and structural responses by mechanotransduction. Cardiac dynamics that are compromised by sarcomeric variants can ultimately lead to cellular remodeling, including changes in nucleus structure, transcription, and gene expression that can possibly support pathological progression.

## 2. Nuclear Components and Mechanosensing

Communication of the cytoskeleton to the nucleus is performed by specific transmembrane proteins located at the nuclear envelope. The nuclear envelope is a double-layered membrane, consisting of inner nuclear membrane (INM) and outer nuclear membrane (ONM), separated by a uniform distance of 50 nm. The actual physical connection of the nuclear envelope and the cytoskeleton is mediated primarily by the linker of the nucleoskeleton and cytoskeleton complex (LINC complex). The LINC complex consists of two proteins: SUN, and nuclear envelope spectrin-repeat proteins (nesprins). SUN and nesprins interact to form a stable “bridge” from the inner nuclear membrane to the outer nuclear membrane. SUN1 and SUN2 isoforms interact directly with nesprins proteins and the nuclear lamina. Nesprins are transmembrane proteins in the outer nuclear membrane that extend into the cytoplasm and connect to intermediate filaments and microtubules, mediating nucleus–cytoskeletal force transmission [23,24].

Variants of SUN1 and SUN2 have been previously reported to be linked to disease pathogenesis, including muscle disorders, cardiac defects, and cardiomyopathy phenotypes [25]. The SUN protein is highly expressed in the nuclear membrane of myocytes and indicated to be vital for nuclear positioning and spacing [25,26,27,28]. The cytoskeleton structural network seems to be significantly susceptible to communication to the nucleus through LINC complex. When variants of SUN are present, myocytes are susceptible to incorrect myonuclear positioning [25,28,29], and defects in muscle function [25,29]. Moreover, patients carrying SUN variants show an abundant loss of nuclear connectivity to the cytoskeleton [25]. This evidence suggests that nuclear positioning defects disrupt nuclear–cytoskeletal coupling, leading to muscular dystrophies. The arrangement of myofibrils is essential for proper muscle contraction, and abnormal nuclear positioning is common in many muscle diseases [30,31].

**Figure 2:**
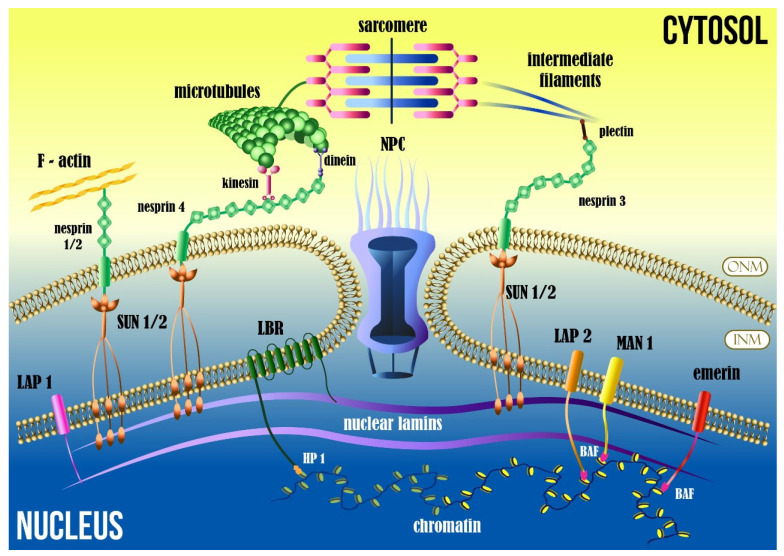
Nuclear envelope and cytoskeleton schematic—Schematic of nucleus’s inner and outer membranes, and nuclear protein interactions with chromatin and cytoskeleton: F-actin, microtubules, and intermediate filaments, showing further connection with the sarcomere. ONM, outer nuclear membrane; INM, inner nuclear membrane; NPC, nuclear pore complex; LAP 1 and 2, lamina-associated polypeptides 1 and 2; LBR, lamin B receptors; BAF, barrier-to-autointegration factor; HP 1, heterochromatin protein 1.

The INM has close contact with the underlying peripheral nuclear lamina and regions of associated chromatin (nuclear lamina-associated domains of chromatin, or LADs). Other associated proteins are part of the inner nuclear membrane, such as emerin [32], lamin B receptors (LBR) [33,34], lamina-associated polypeptides 1 and 2 (LAP1 and LAP2) [35,36,37,38], MAN1 (LEMD3) [39], and nuclear envelope spectrin-repeat proteins (nesprins 1, 2, 3, and 4) [40,41] (Figure 2). They are known to bind to the lamina, provide structural integrity, and communicate mechanical cues, thereby participating in signal transduction pathways. Variants of proteins such as emerin, nesprin-1 and 2, lamin A/C, and LAP1 and LAP2 have been reported to be associated with pathological cardiac development and muscular dystrophy [24,29,42,43,44,45]. The communication of cytoskeleton tension forces is therefore supported by the nuclear envelope proteins, which is particularly important within myocytes.

The nuclear lamina is a dense meshwork of proteins located adjacent to the interior side of the INM. The meshwork consists mainly of aggregated arrangement of filament lamins [46]. The A-type lamins, lamin A/C, are found in the nuclear periphery and nuclear interior, while the B-type lamins, lamin B1 and B2, are concentrated in the nuclear periphery [47]. The function of the nuclear lamina is to provide a structural (mechanical) framework for the nucleus [48], and assist other functions in the nucleus morphology, such as nucleoplasmic reticulum formation [49,50], support chromatin organization by defining territories within the nucleus and directly tethering LADs to the nuclear envelope [51,52,53,54], support the mitosis cycle by regulating mitotic spindle assembly and positioning [55,56], participate in DNA replication with focal replication regions in early S-phase [57,58], and regulate gene transcription through interactions with transcription factors, such as barrier-to-autointegration factor (BAF), that directly binds to DNA and it is involved in transcription control at an epigenetic level [59,60,61].

The intrinsic lamin–chromatin interaction leads to response of DNA regulation, chromatin organization, and gene transcription [62,63]. Disease-associated variants of genes encoding proteins of nuclear lamina are classified as laminopathies. Laminopathies can be associated with clinically distinct pathological processes in skeletal and striated muscle as in Emery–Dreifuss muscular dystrophy, fat distribution as in Dunnigan-type lipodystrophy, craniofacial abnormalities seen in mandibuloacral dysplasia, accelerating aging disorders such as Hutchinson–Gilford progeria syndrome, and electrical and mechanical dysfunction of the heart reported in dilated cardiomyopathy [64,65,66]. A study on lamin A/C haploinsufficiency in mouse hearts showed that pressure overload presents potential relationship with transcription factors that suppress mechanosensitive genes in response to reduce the physical cues in the nucleus [67]. The suppression effect of lamins is reported to be associated to interactions with transcription factors of the activator protein-1 (AP-1) family [68], and defective nuclear morphology, such as elongated nucleus and irregular chromatin, and heterochromatin clumping are found to be significantly related to lamin A/C variants [69]. Moreover, lamin A/C also plays a role in reinforcing the nucleus overall stiffness in order to protect nuclear homeostasis when under physical strain [70,71].

Interestingly, the disruption of SUN1 protein has been reported to suppress pathological progression of cardiomyopathic phenotype. By potentially decreasing the mechanoresponsive effects of the LINC complex and reducing overall tension forces in the nucleus, the SUN1 disruption increases the lifespan of cardiomyocyte through the protection of nuclear homeostasis [72]. Additionally, similar nuclear responses can be achieved by the SUN2 protein when human mesenchymal stem cells (hMSCs) are grown on increasingly stiff substrates [73]. The SUN2 decouples itself from the nuclear membrane, consequently “breaking the bridge” of nuclear and cytoskeleton mechanotransduction, conferring protection to the nucleus and preventing DNA damage [73].

Communication between the cytoskeleton and nuclear envelope has been shown to play a crucial role in physical nuclear remodeling, gene expression, and to be involved in multiple pathogenesis processes. As the nuclear envelope proteins can sense the propagation of mechanical stimuli from dynamic intermediate filaments, F-actin and microtubules can rapidly respond to force [5]. The nucleus is 2 to 10 times stiffer than the cytoplasm, and nuclear deformability can be a rate-limiting factor during cell migration [74,75]. Studies with cells lacking nuclear envelope proteins such as emerins [76], nesprins 1 and 2 [77], or SUN1 proteins [78] show decreased cellular migration, and reduced levels of lamin A/C expression have been associated with increased nuclear deformability [79].

A few examples of mechanical forces that can lead to physical nuclear remodeling are: cell migration, shear stress, cellular compression, and contraction. Cells must deform to fit through available space when migrating. Endothelial cells that line blood vessels normally experience shear stress associated with physiological blood flow [80]. These cells are constantly under fluid shear stress, a physical force that modulates endothelial cell function with changes in blood flow due to altered cardiac output, blood pressure, etc. [81]. The presence of shear stress leads to nuclear flattening in endothelial cells, displacing the nucleus downstream of flow, increasing nuclear length and stiffness in a matter of a few hours [80]. Compression of the nucleus due to cell contraction also has shown that the nucleus undergoes significant changes in shape, volume, and morphology in chondrocytes and myocytes [82,83,84]. Alternatively, the same effect should not be different from mechanical forces that the nucleus must withstand in cardiomyocytes. The consistent nature of contraction–relaxation cycles should lead to similar nuclear responses in cardiomyocytes to provide nuclear protection.

The stiffness of the nucleus is clearly important for determining the amount of compression, but both the forces generated by and the stiffness (compliance) of the contractile components, relative to that of the nucleus, will be important on their own [85,86,87,88], as well as for mechanotransduction. Correlations of nucleus remodeling and cellular stress have shown mounting evidence of the nucleus’s dynamics under physical and environmental stress, and its mechanosensitive capacity [89]. Conversely, both the lamina meshwork and chromatin condensation are key contributors to protect the nuclear envelope and avoid DNA damage [7,90].

## 3. Sarcomere Contraction and Sarcomeric Cytoskeleton

The sarcomere is the defining structural feature and the basic functional unit of striated muscles including the cardiac muscle. It is a highly organized array of myofilament proteins (Figure 1) that are responsible for active forces and muscle shortening [91,92]. The sarcomere consists of thin filaments, thick filaments, and titin filaments that have been referred to as the third filament system, although titin interacts with both thin and thick filaments. The thin filaments consist of actin monomers, troponin complex, and tropomyosin [93,94]. Each thick filament consists of a bipolar assembly of myosin molecules. Myosin motor domain “heads” that can form cross-bridges with actin are prominent features of both ends of the thick filament, while a central bare zone has no heads and only contains myosin rods that form the filament’s backbone [95,96]. The sarcomere is divided into different regions. Two Z-discs form the boundaries of a sarcomere with each Z-disc located in the middle of the I-bands (isotropic bands) of adjacent sarcomeres. Only thin filaments are present in the I-bands, with the thin filaments on each side of a Z-disc having opposite structural polarity. Thick filaments define the A-bands (anisotropic bands), which, at physiological sarcomere lengths, also contain an overlap zone where thin filaments interdigitate between thick filaments and where cross-bridges can form [97]. The H-zone is the central portion of the A-band where only thick filaments are present. The M-line is the center of the sarcomere structure and derives from proteins that connect thick filaments at the middle of the bare zone [98,99].

The troponin complex on thin filaments is a fundamental Ca^2+^-regulatory protein complex that is necessary for muscle activation. The complex consists of three subunits, troponin-C (TnC), troponin-I (TnI), and troponin-T (TnT). The subunit TnC is responsible for specific Ca^2+^ binding [100], where cytosolic cation Ca^2+^ is able to bind [101,102,103]. The subunit TnI inhibits interactions of thick and thin filaments. TnI has an interesting “connecting” role that makes it central to the troponin core domain: part of TnI forms an α-helical coiled-coil with TnT, while another part of TnI binds to the C-terminus of TnC [94,104,105]. In addition to these stable interactions that hold together the three subunits of a troponin molecule, TnI has a flexible C-terminus that alternatively interacts with actin and tropomyosin at relaxing/diastolic levels of cytoplasmic Ca^2+^, or the N-terminus of TnC when activating Ca^2+^ is bound to the site II EF-hand in TnC’s N-lobe [94,105,106,107,108,109]. The inhibitory domain of TnI is a key portion of TnI’s C-terminus [110].

During activation, the cytoplasmic Ca^2+^ transient leads to the movement of TnI’s C-terminus from actin and tropomyosin as the TnI switch peptide sequence binds TnC’s N-lobe. The associated structural changes in troponin lead to displacement of tropomyosin [111,112]. Subunit TnT is the largest subunit of the troponin complex [113]. It anchors the troponin complex to tropomyosin [94,105,114,115]. Each tropomyosin molecule is a dimer where the two polypeptides form an α-helical coiled-coil [116,117]. Tropomyosin’s persistence length (L_p_), which is a measure of the molecule’s flexibility, is slightly longer than the length of an individual molecule [115,116,117]; this degree of flexibility has significant implications for tropomyosin function in the thin filament. Tropomyosin molecules bind end-to-end, or head-to-tail [93,118], such that there are two strands along the length of a thin filament. The structural regulatory units consist of one tropomyosin molecule, one troponin molecule, and seven actins [119] assembled into a structure that has been deduced by and is being further refined by cryo-electron microscopy [93,94,105].

The myosin molecules that comprise the bulk of thick filaments each consist of a pair of myosin heavy chains (MHC) and two pairs of myosin light chains (MLC), the essential light chain (ELC) and regulatory light chain (RLC) [120,121]. Each MHC’s N-terminus forms the motor domain (head) that contains an actin-binding site and a nucleotide-binding site [96,122,123]. The motor domain is connected to the α-helical C-terminus via a lever arm that includes two MLCs. A myosin molecule forms when the C-termini of two MHCs generate an α-helical coiled-coil tail; the tails from approximately 300 myosin molecules come together to form the backbone of a thick filament [124]. The mechanical force required for muscle contraction is generated by cyclic interactions between myosin motor domains of thick filaments and exposed myosin-binding sites on the actin of thin filaments. Association and dissociation of these proteins is called the cross-bridge cycle, a well-accepted theory of muscle contraction [125].

Cross-bridge cycling is powered by ATP hydrolysis by myosin motor domains and is regulated by Ca^2+^ binding to troponin [126]. A myosin head hydrolyzes a molecule of ATP (specifically MgATP, the noncovalent complex of Mg^2+^ and ATP) at the nucleotide-binding site, which alters the cross-bridge structure by tilting the head relative to the lever arm into a pre-force generating position. The ATP molecule is hydrolyzed into ADP and inorganic phosphate (Pi). Myosin heads in the disordered relaxed state—with ADP and Pi at the nucleotide site—can form a weak, electrostatic interaction with an available actin in a manner that is consistent with diffusional limitation [127,128,129,130,131,132]. The myosin associated with actin undergoes a weak-to-strong conformational change—the power stroke—by tilting of the head relative to the lever arm around the hinge. This structural change is closely associated with Pi dissociation [133], and is transformed into mechanical force that leads to the thin filament sliding past the thick filament towards the center of the sarcomere, causing shortening of the sarcomere [134]. Following the power stroke, ADP dissociates from the myosin head and, at physiological ATP levels, is rapidly replaced by ATP leading to the dissociation of the myosin head from actin [135,136]. The velocity of sarcomere shortening varies with the external load, with this force–velocity relationship in the form of a rectangular hyperbola [137,138]. The myosin head, unbound from actin, can now hydrolyze ATP, and initiate another cross-bridge cycle.

The sarcomeric structure is remarkably distinguished by its transverse axial order of thin and thick filaments forming the myofilament lattice [136]. The lattice space maintains a coordinated landscape of filaments arrangement for the contractile performance and force production [139]. Active tension generated by cross-bridge cycling is transmitted through thin filaments to the Z-discs at the boundaries of the sarcomere. The Z-disc contains a scaffold of crosslinking proteins that anchors the thin filaments, participates in tension transmission along myofibrils, and properly maintains myofilament lattice dimensions of the sarcomere [140]. In cardiac muscle, the thin filaments are connected through the N-terminus of the protein α-actinin 2 which contains an actin-binding domain (ABD). The adjacent neck regions of the α-actinin 2 grant flexibility to form an anti-parallel conformation with central rod-shaped regions of spectrin repeats [141]. The C-terminus region consists of EF-hand motifs with a calmodulin-like domain (CAMD) [142]. Whereas the CAMD is Ca^2+^ sensitive in non-muscle isoforms, muscle isoforms have lost their ability to bind to Ca^2+^ [141].

Along with actin interaction, α-actinin binds to the giant protein titin (formerly known as connectin). Titin is such a large protein that its filaments extend the entire length of a half-sarcomere, connecting from the Z-disc at one sarcomere boundary to a thick filament up to the M-line in the middle [143]. Therefore, only part of titin comprises within the Z-disc [144,145]. A series of Z-repeat copies are present in the Z-disc region of titin and have been reported to be the connectors to α-actinin CAMD domain [144,146,147,148]. The N-terminus of titin is ultimately anchored to the Z-disc by the interaction of telethonin (also known as T-cap) [149,150,151], forming a complex that interacts with other ligand proteins, conferring the bridging structure between sarcomeres [150,151]. The main roles of titin in sarcomere contraction are not only its mechanical elastic component and contribution to passive force, but also to hold a stable sarcomeric structure, withstanding continuous compressive forces in the cardiac muscle [149,152].

Another notable protein that assists in determining characteristic features of the sarcomeric cytoskeleton is nebulette. Nebulette is a cardiac-specific sarcomeric isoform of the longer nebulin protein that is predominantly expressed in skeletal muscle. Nebulin plays an important role in maintaining sarcomere length, and both have significant role in supporting Z-line integrity [153,154], a crucial attribute for force generation in striated muscle [151,153,155]. The skeletal muscle protein nebulin is a giant protein that extends the entire thin filament length. However, the cardiac muscle protein nebulette covers only a short length of the thin filament and it is localized at the Z-disc. Both isoforms display interactions in the C-terminal region with other Z-disc proteins such as α-actinin 2 [156], myopalladin [157], and titin [156,158]. While the N-terminal region of nebulin interacts with tropomodulin (T-mod) at the pointed end of the thin filament [158,159,160,161], the nebulette N-terminus may show interactions with the protein filamin-C [162], but the region has not yet been fully characterized, whereas central domains are known to interact with actin, tropomyosin, and troponin [162,163]. The Z-disc-associated proteins are an important scaffold which provide the structure necessary for sarcomeric lattice regulation and integrity, which are crucial for protein interactions and signaling functions [158,159,160,161,164].

The localization of desmin filament networks anchoring to sarcomeric proteins at the periphery of the Z-discs seems to be the key feature of the sarcomeric cytoskeleton connection to other organelles [165]. While the key mechanosensory pathways of sarcomere and nucleus are not yet fully understood, it is possible to deduce which proteins would act as players in mechanotransduction by the neighboring interactions they make [166]. Since the Z-discs connect directly to the intermediate filaments of desmin (Figure 1), this region is certainly a pivotal mechanotransduction pathway. Recent studies have shown that desmin contains interacting sites with nebulin in skeletal muscle [167,168] and nebulette in cardiac muscle [169]. The connections of Z-disc proteins with the intermediate filaments of desmin is the final puzzle piece for mechanosensing and mechanotransduction of sarcomere tension to the nucleus [170]. Variants of sarcomeric proteins lead to the impaired contractility of cardiomyocytes [17,93,139,171,172,173], this impaired contraction–relaxation cycles are sensed and transmitted through Z-discs to the cytoskeleton. Thus, sarcomeres that display overloaded or insufficient contractility will transmit differential tension to the nucleoskeleton, inducing nuclear responses.

## 4. The Cytoskeleton Network

Force generated by the sarcomere propagates longitudinally and laterally [174]. The lateral component of force generated by cross-bridges and lateral communication of the Z-discs with integrins and dystroglycan protein complexes are responsible for propagation of force to the extracellular matrix and sarcolemma [175]. Dystroglycan variants are largely associated with muscle dysfunction and dystrophy, and the sarcolemma plays a crucial role in cardiomyocyte communication and adhesion [176]. Longitudinal communication between adjacent sarcomeres in a myofibril consists of the specialized connections via Z-discs, such as the large polypeptide titin chain and α-actinin 2 [177], and cytoskeletal elements, such as intermediate desmin filaments and microtubules [178,179], which are responsible for cellular architecture, tension homeostasis, and propagation of mechanical signals throughout the cytoskeleton, affecting other organelles, including the nucleus [3].

The cytoskeleton consists of a dense network of filamentous actin, microtubules, and intermediate filaments [180], and it provides support for mechanical integrity, morphology sustenance, and cellular shape, and more importantly: a structural nuclear protection to the genome [7,181,182]. The filaments of actin in the cytoplasm are expressed in different isoforms from the thin filaments in the sarcomere [183,184]. While α-actin is predominantly expressed in adult cardiomyocytes, β-actin and γ-actin are present in lower levels in the cytoplasm [185,186]. The cellular actin network and its actin isoforms promote architectural support, facilitate cellular dynamics and mechanics, and assist functions such as adhesion, migration, and contractility [183,187]. Cytoplasmic actin can transmit mechanical force by signaling cues from the ECM through integrins, dictating cell stiffness and cell adhesion [185,188].

Microtubules form a dense network assembled by tubulin isoforms. It has been described that cardiomyocyte microtubules form three dynamic lattices in the cell: along the long axis and closely associated to mitochondria, around the nucleus, and along the short axis [189]. Being the stiffest component of the cytoskeleton, it evolves in postnatal development of the heart, and provides resistance upon cardiomyocyte diastole and systole loads [189,190]. Microtubules not only perform a cellular structural role, but cargo transport as well. Motor proteins, such as kinesin and dynein, can travel along the microtubule filaments carrying valuable cargo such as mRNA and ribosomes [189,191,192]. As part of the mechanosensing pathway, microtubules can contribute to stress and cardiac remodeling through post-translational modifications (PTMs) [189,190]. Studies have reported that microtubule acetylation can alter the filament spacing, increasing the ability to bend, and therefore withstand higher compression burden [193]. Additionally, microtubule detyrosination can crosslink with intermediate filaments of desmin at the Z-disc region in order to resist contraction loads [189,190,194].

Different from microtubules and actin filaments, the intermediate filaments are composed of four different types of protein filaments. The myocytes can only express the filament types III, IV, and V [178,195]. The desmin filament (type III) is specific to muscle cells, the most abundant type, and is the major intermediate filament linked to cardiomyopathies and heart failure [178]. Intermediate filaments form an intracellular meshwork, connecting Z-discs, sarcolemma, mitochondria, intercalated discs (ICDs) and desmosomes, and nuclei [195]. Cytoskeletal variants can lead to broad responses in cellular dysfunction and structural alterations. Desmin variants have been associated with defective contractility mechanics and cellular remodeling [6,196,197]. Desmin knock-out has shown evidence of structural destabilization of the cytoskeleton and mitochondrial dysfunction in cardiomyocytes [198]. Altered microtubule dynamics resulting from reduced detyrosination of tubulin have been associated with reduced contractility and loss of its viscous resistance in cardiomyocytes. The viscoelastic resistance change impacts overall cardiac performance [190]. Increased viscous resistance, or stiffness, has been reported in cardiomyocytes of failing hearts [199], and ventricular hypertrophy has been associated to microtubule stabilization [179,200].

With such consolidated networks, these three cytoskeleton elements act as dynamic bridges to intracellular hubs that can interact and transmit information. The cytoskeleton proteins are crucial players in mechanotransduction pathways. The mechanosensing proteins in membranes sense and transmit the biochemical signals mediated by the cytoskeleton. The associated proteins of nuclear envelope membrane, such as LINC complex and nesprins, are the immediate scaffold of mechanotransduction signaling to the nucleus. Thus, an abundance of evidence points to the cytoskeleton network as a connecting ”bridge” to the sarcomere, which is a key site for mechanotransduction in muscle cells [201].

## 5. Mechanotransduction in Cardiomyopathy

A significant proportion of cardiomyopathies are strongly associated to inherited or acquired sarcomeric variants [202,203,204,205,206]. These variants can lead to impaired sarcomeric function and contractility dysfunction in cardiomyocytes. Pathogenic variants in myofilament protein genes such as β-myosin heavy chain (*MYH7*) [207], myosin-binding protein C (*MYBPC3*) [207,208], and troponin T (*TNNT2*) [209,210] are frequently found in hypertrophic cardiomyopathy (HCM) and dilated cardiomyopathy (DCM) cases [208]. At a molecular scale, the sarcomeric dysfunction induces disease pathogenesis and maladaptive responses in cardiac muscle [211], such as altered calcium handling [212,213], fibrosis [214], arrhythmias [215], and hypertrophy [216]. The activation of maladaptive cardiac remodeling is a response to faulty contractility which is biochemically transmitted through protein interactions [3,217].

Mechanotransduction describes processes in which mechanical forces are “translated” into biological responses [218]. An important example of mechanosensitive proteins is the family of Piezo channels. Piezo 1 and Piezo 2 are cation channel mechanoreceptors that are activated by the stretching of the membrane in which they reside, transducing the mechanical signal into an electrical signal [218,219]. Mechanotransduction is critical for proper biological function, while abnormal mechanotransduction can lead to a variety of disease processes [220], including prolonged excessive mechanical stimulation [221]. As cells can sense mechanical cues from the environment and transmit biochemical signals through the cytoskeleton [222], the mechanical forces generated from the sarcomere can also be sensed and transmitted intracellularly. When the contractile apparatus—the myofilaments of the sarcomere—exerts an overload of tension inside the cells, the excessive signal will be perpetuated to other organelles, such as the nucleus. For example, altered Ca^2+^ sensitivity can easily change contraction–relaxation cycles in cardiomyocytes, an overloaded contractility, such as may occur in HCM, will be transmitted through the cytoskeleton. The nucleus would need a mechanosensitive response to the contractile burden to attempt to maintain nuclear (and possibly also cellular) homeostasis.

Many studies of the propagation of mechanical stimuli in fibroblasts, myoblasts, and epithelial and cancer cells have been published [25,223,224,225], yet little is known about the mechanotransduction of sarcomeric forces to the nucleus in cardiomyocytes. This is significant considering that HCM variants are typically associated with greater contractility by increased myofilament Ca^2+^ sensitivity [21,172,173,226,227] and, conversely, DCM variants are associated with lower contractility (e.g., by reductions of Ca^2+^ affinity) and ATPase activity [21,171,172,173,227,228,229]. It is expected that mechanical signals are directly transmitted from the sarcomere to the nucleus [230]. The forces sensed by the nucleus can induce cellular adaptations, such as alterations in lamina levels, localization of transcription factors, chromatin organization, and changes in gene expression [5,201]. Transcription regulator Yes-associated protein (YAP) together with transcriptional coactivator with PDZ-binding motif (TAZ), or YAP/TAZ, is one example of the signaling pathways that are sensitive to the mechanical cues from the ECM [231,232]. Regulation of YAP/TAZ activity is dependent on physical stiffness and mechanical load induced by the ECM, whereas cytoskeleton tension leads to nuclear translocation and activation of YAP/TAZ. Active dephosphorylated YAP/TAZ localized in the nucleus bind to TEA domain family member (TEAD) transcription factors, which can transcribe genes that control cell proliferation, apoptosis, and cell fate [231,232,233,234]. In the heart, YAP/TAZ activation has been shown to be essential for cardiac regeneration after myocardial infarction [235], where YAP nuclear translocation promotes cardiomyocyte proliferation. Moreover, this regulation of proliferation by YAP can be modulated by β-blocker treatment through the RhoA GTPase axis [236], which known to regulate cellular contractility through the activation of ROCK and myosin. Moreover, a proteomics study has also shown the regulation of a subset of proteins in cardiomyocyte, such as collagen I and cardiac-specific excitation–contraction proteins, by the modulation of ECM stiffness [237].

Additionally, to counterstrike the compression tension, the nucleus can increase or decrease its stiffness to prevent possible deformability [238,239], since nuclear deformations and severe altered morphology can lead to nuclear dysfunction by loss of nuclear compartmentalization, or decreased transcription activity and nuclear rupture [239]. The stiffness of the nucleus is associated to variations in nuclear lamin A levels [240], and chromatin condensation [241], which have been reported to closely assist nuclear plasticity and sensitivity to strains [90,242]. A study reported different levels of lamin A and its transcription levels in various tissue stiffness, altering their own nuclear stiffness in response to different intracellular tensions [243]. Decreased levels of lamin A are insufficient to protect the nucleus from mechanical stress, leading to excessive deformation and disruptions on the chromatin [243,244]. While the lack of lamin A can cause DNA damage, nuclear rupture, and loss of nuclear repair factors [7], the expression of lamin A suggests overall protection of the genome [5,7]. *LMNA* gene promoter contains a novel retinoic acid (RA)-responsive element (RAR) that binds to transcription factors [245]. RAR can regulate lamin A expression downstream from mechanical tension, but lamin A also regulates nuclear translocation of RA receptor by other factors such as polymerase I (Pol-I) and transcript release factors (PTRF) which is downstream of lamin A. These findings suggest lamin A positively regulates its own transcription and its regulation can happen simply by mechanoregulation under varied stress, strain, or stiffness [243]. Moreover, the phosphorylation of lamin A/C that promotes the degradation of the protein is also regulated by the tensional stress acting on the nucleus. High-tension stress on the lamina will lower kinase accessibility and inhibit phosphorylation, hence causing a slower turnover, suggesting another role of mechanical cues in nuclear remodeling [246].

Lamins interact with chromatin at the nuclear periphery via LADs and the protein LAP2 (Figure 2), which play a role in chromatin organization, as well as maintenance of active/inactive chromatin and transcriptional states [71,247]. Lamin A/C has a dynamic structural, tension-sensitive effect on chromatin and its conformation [201]. Chromatin is not homogeneous. Transcriptionally inactive heterochromatin is located more at the periphery of the nucleoplasm, while the transcriptionally active, gene-rich euchromatin is located at the nuclear interior. Post-translational modifications can differentially organize the DNA into condensed or decondensed regions, and the level of chromatin condensation can not only influence the gene expression, but also the mechanical properties of the nucleus [201]. Rapid mechanosensitive responses are associated with post-translational modifications (PTMs) in chromatin [248]. Loss of HDAC3 (histone deacetylase 3) and the activity of transcription factor MEF2 (myocyte enhancer factor-2) has been shown to be associated to cardiac hypertrophy [249]. Mechanical forces induce chromatin condensation and increase lamin levels, which prevent nuclear deformation and DNA damage [247]. This leads to altered gene expression [14], interference with multiple signaling processes, and the development of pathology and disease progression of cardiomyopathic patients. In fact, laminopathies are associated variants of LMNA-associated heart diseases which express the detachment of heterochromatin from the nuclear lamina, inducing changes in chromatin condensation that are correlated to alterations in gene expression [250].

Heart tissue mechanics are major contributors to cellular mechanical tension. Cardiomyopathy phenotypes show altered excitation–contraction coupling at the cellular level, and the heart therefore presents irregular or inefficient electrical action potential propagation. Proper sarcomeric contraction and relaxation are crucial for the heart’s beating rate, synchronicity, and force-frequency propagation [251]. The impaired contractility found in cardiomyopathic myocytes may induce changes in the nucleus by the process of mechanotransduction. Mechanical sarcomeric forces have a critical link through the Z-disc and desmin filaments to the nucleus. Nucleus remodeling by mechanical strain in mammalian cells is largely described in the literature. By the incessant contraction–relaxation nature of cardiomyocytes, significant nuclear alterations such as lamin A/C expression levels and chromatin condensation are expected, consequently changing the overall nuclear stiffness of nuclear membrane and—possibly harmful—gene expression changes [252].

## 6. Conclusions

This review summarizes the importance of the further investigation of mechanotransduction signals and nucleus remodeling in cardiomyocytes in health and disease. Although many published studies focus on the effects of sarcomeric components and variants in cardiomyopathies, little is known about intracellular maladaptation caused by the impaired contractility of sarcomeres. Cardiomyocytes are under constant physical contractile activity, generating tension that activates mechanosensitive pathways, which lead to mechanoresponsive effects that may ultimately become maladaptive in cardiomyopathies. This brings attention to the need for the meticulous investigation and clarification of nuclear mechanotransduction pathways in cardiomyopathy models.

## Data Availability

Not applicable.
